# Intracortical and Intercortical Motor Disinhibition to Transcranial Magnetic Stimulation in Newly Diagnosed Celiac Disease Patients

**DOI:** 10.3390/nu13051530

**Published:** 2021-05-01

**Authors:** Francesco Fisicaro, Giuseppe Lanza, Carmela Cinzia D’Agate, Raffaele Ferri, Mariagiovanna Cantone, Luca Falzone, Giovanni Pennisi, Rita Bella, Manuela Pennisi

**Affiliations:** 1 Department of Biomedical and Biotechnological Sciences, University of Catania, Via Santa Sofia 97, 95123 Catania, Italy; drfrancescofisicaro@gmail.com (F.F.); manuela.pennisi@unict.it (M.P.); 2 Department of Surgery and Medical-Surgery Specialties, University of Catania, Via Santa Sofia 78, 95123 Catania, Italy; pennigi@unict.it; 3 Department of Neurology IC, Oasi Research Institute-IRCCS, Via Conte Ruggero 73, 94018 Troina, Italy; rferri@oasi.en.it; 4 Gastroenterology and Endoscopy Unit, University Hospital “Policlinico G. Rodolico-San Marco”, Via Santa Sofia 78, 95123 Catania, Italy; dagate@policlinico.unict.it; 5 Department of Neurology, Sant’Elia Hospital, ASP Caltanissetta, Via Luigi Russo 6, 93100 Caltanissetta, Italy; m.cantone@asp.cl.it; 6 Epidemiology and Biostatistics Unit, Instituto Nazionale Tumori-IRCCS “Fondazione G. Pascale”, Via Mariano Semmola 53, 80131 Napoli, Italy; l.falzone@istitutotumori.na.it; 7 Department of Medical and Surgical Sciences and Advanced Technologies, University of Catania, Via Santa Sofia 87, 95123 Catania, Italy; rbella@unict.it

**Keywords:** gluten-related pathology, cortical excitability, transcallosal inhibition, transcranial magnetic stimulation, executive dysfunction, gamma-amino-butyric acid

## Abstract

Background: Celiac disease (CD) may present or be complicated by neurological and neuropsychiatric manifestations. Transcranial magnetic stimulation (TMS) probes brain excitability non-invasively, also preclinically. We previously demonstrated an intracortical motor disinhibition and hyperfacilitation in de novo CD patients, which revert back after a long-term gluten-free diet (GFD). In this cross-sectional study, we explored the interhemispheric excitability by transcallosal inhibition, which has never been investigated in CD. Methods: A total of 15 right-handed de novo, neurologically asymptomatic, CD patients and 15 age-matched healthy controls were screened for cognitive and depressive symptoms to the Montreal Cognitive Assessment (MoCA) and the 17-item Hamilton Depression Rating Scale (HDRS), respectively. TMS consisted of resting motor threshold, amplitude, latency, and duration of the motor evoked potentials, duration and latency of the contralateral silent period (cSP). Transcallosal inhibition was evaluated as duration and latency of the ipsilateral silent period (iSP). Results: MoCA and HDRS scored significantly worse in patients. The iSP and cSP were significantly shorter in duration in patients, with a positive correlation between the MoCA and iSP. Conclusions: An intracortical and interhemispheric motor disinhibition was observed in CD, suggesting the involvement of GABA-mediated cortical and callosal circuitries. Further studies correlating clinical, TMS, and neuroimaging data are needed.

## 1. Introduction

Within the wide spectrum of gluten-related disorders [1], it is now established that the classic celiac disease (CD) is only the tip of the “CD iceberg” [2], since from five to six-fold more subjects exhibit non-typical phenotypes [3]. As such, CD is currently viewed as a multiorgan disease with multifactorial pathogenesis and clinical manifestations.

Among extraintestinal features, neurological and neuropsychiatric manifestations are still a diagnostic challenge in CD, given that they can precede or follow the disorder or be already evident at the onset [1,4,5,6]. In a recent cohort prospective investigation of newly diagnosed subjects of CD [7], neurological deficits were common, and a significant volume decrease in some cerebral regions, with transglutaminase (TG)-6 autoantibodies, was observed. These findings highlight the importance of prompt diagnosis, awareness among physicians, and compliance to an adherent gluten-free diet (GFD) to prevent, or at least limit, the neurological involvement and related disability [7]. It has been also demonstrated that most of the subjects with confirmed CD referred for neurological consultation already show changes at brain magnetic resonance imaging (MRI) [8]. Based on these considerations, a reliable diagnostic tool, able to early detect process, progression, and complications underlying the disease, as well as the response to the GFD, is needed.

Within the neurophysiological techniques, motor evoked potentials (MEPs) to transcranial magnetic stimulation (TMS) are among the electrophysiological methods that can non-invasively probe the state of excitation of cortical motor areas in vivo [9] and the conduction along the cortico-spinal pathway [10], as well as the functional connectivity across hemispheres [11]. TMS is also able to unveil preclinical motor impairment in several neurological, psychiatric, and some secondary diseases of the central nervous system (CNS), also providing prognostic [12] and therapeutic implications [13]. Finally, the “pharmaco-TMS” may distinctively explore various transmission pathways within the CNS, such as that mediated by gamma-aminobutyric-acid (GABA), glutamate, acetylcholine, and monoamines, by administering drug agonists or antagonists [14,15,16].

To date, only a few studies have applied TMS in CD. In 1999, Pellecchia and colleagues first reported a decreased MEP size in the rectus femoris muscle in a CD patient, who improved after the GFD [17]. A year later, a delayed MEP in the left tibialis anterior muscle and a change of cortical inhibition was reported in one of three CD subjects with cortical myoclonus [18]. More recently, systematic studies before and after GFD have specifically evaluated the TMS profile of cortical excitability in CD.

In the first study, twenty de novo subjects without clinical involvement of the CNS and twenty controls matched for age were included [19]. TMS showed a hyperfacilitation and a disinhibition of the primary motor cortex (M1) in patients, suggesting an impaired glutamatergic and GABAergic circuitry, respectively. Unbalanced inhibitory and excitatory transmissions within the M1 was hypothesized as the correlate of a cross-interaction between some neuronal antigens and gliadin antibodies. Alternatively, the deposition of tissue TG-immunoglobulin might lead to a pathological ion concentration at the level of neuronal membranes. Similarly, the CNS-produced antibodies against glutamic acid decarboxylase may impair the activity of GABAergic interneurons [19].

The same sample was re-assessed following a relatively short-term GFD (median 16 months) [20]. Gastrointestinal manifestations improved although, unexpectedly, the excitation state of their M1 to TMS enhanced further. This result was thought to be an index of an adaptive re-modeling of the motor areas, probably not related to the GFD. It is also reasonable to hypothesize that the duration of the diet or its adherence was not enough to produce a significant recovery [20]. A further study following a substantially longer gluten restriction (mean period 8.35 years) revealed that only a sustained GFD could restore the TMS-associated modifications in adults with CD. However, some excitatory changes seems to persist, likely indicating a synaptic intracortical rearrangement of the “celiac brain”, mostly involving the glutamate-mediated interneurons [21].

Beside the intracortical excitability, to date, no data are available on the intercortical excitability in CD. Namely, the interhemispheric motor functioning to TMS, as indexed by specific measures of transcallosal inhibition, has never been investigated in these patients. In this cross-sectional study, we aim to evaluate the callosal function to TMS in de novo, neurologically asymptomatic, CD patients compared to healthy controls. We hypothesized that these subjects, as already observed for the measures of intracortical excitability, might exhibit changes in intercortical excitability, even at a subclinical level.

## 2. Materials and Methods

### 2.1. Participants and Evaluation

Fifteen consecutive de novo subjects with CD (13 women; mean age ± standard deviation (SD): 34.10 ± 12.03 years), diagnosed according to the European Society for Pediatric Gastroenterology Hepatology and Nutrition guidelines [22], were enrolled from the Regional Center for Celiac Disease of the Azienda Ospedaliero-Universitaria “Policlinico G. Rodolico-San Marco” of Catania (Italy). Fifteen healthy individuals (12 women; mean age ± SD: 34.90 ± 9.18 years), matched for age with patients, served as the control group. All patients were on free diet at the time of the enrolment.

Criteria of exclusion were: age < 18 years; CNS (i.e., Parkinson’s disease, stroke, dementia, traumatic brain injury, multiple sclerosis (MS), epilepsy, etc.) or psychiatric diseases (major depressive disorder (MDD), bipolar disorders, schizophrenia, obsessive–compulsive disorders, etc.); chronic, acute, or uncompensated medical conditions (i.e., heart failure, coronary heart disease, liver or kidney failure, etc.); illicit drug abuse or alcohol dependency; intake of drugs influencing mood or M1 excitation state (i.e., antidepressants, benzodiazepines, mood stabilizers, neuroleptics); pacemaker, pregnancy, or other conditions precluding MEP, according to the latest guidelines on TMS safety [23].

The clinical-demographic assessment consisted of: age, sex, educational level, handedness, general and neurological exams, co-morbidities. A screening test of global cognitive status by means of the Montreal Cognitive Assessment (MoCA), adjusted for age and educational level for each individual [24], and a symptom estimation of depression through the 17-item Hamilton Depression Rating Scale (HDRS) [25] were performed by an operator (M.C.) blind to the participant status as patient or control. Additionally, a computed tomography (CT) of the brain was acquired in all patients with a helical 64-slice General Electric scanner, with 2.5 mm slice thickness, in order to properly detect intracranial calcifications (that can be found in CD) and to exclude clear neuroradiological lesions.

The Ethics Committee of the Azienda Ospedaliero-Universitaria “Policlinico G. Rodolico-San Marco” of Catania (Italy) approved the study (code of approval: Prot. n.103/694). Informed consent was signed by each individual prior to participation in accordance with the Declaration of Helsinki in 1964 and subsequent amendments. Every procedure was carried out in a dedicated laboratory by experienced operators.

### 2.2. TMS Procedures

TMS was carried out by means of a high-power Magstim 200 stimulator (Magstim Co., Whitland, Dyfed, UK). A 70 mm figure-of-eight coil was positioned on the M1 of the dominant hemisphere at the best position of the scalp to evoke MEPs in the first dorsal interosseous (FDI) muscle of the contralateral side, according to the Edinburgh Handedness Inventory (EHI) [26]. Electromyography (EMG) was performed with silver/silver-chloride disposable self-conductive and self-adhesive surface electrodes. The active electrode was positioned on the belly of the target muscle (FDI), the reference at the metacarpal–phalangeal joint of the index finger, whereas the ground on the wrist dorsal surface. For the conduction study of the motor nerve, i.e., compound motor action potential (CMAP) and F-waves of the ulnar nerve, a bipolar nerve stimulation electrode, with an interelectrode separation of 25 mm and 6-mm diameter felt pads, was used while recording from the target muscle (FDI).

The resting motor threshold (rMT) was considered as the minimum intensity of stimulation capable to induce, at rest, a MEP of an amplitude >50 µV in five of ten trials, as recommended by the international guidelines [27]. The central motor conduction time (CMCT) was estimated by subtracting the time of conduction along peripheral nerves, calculated with the F-wave technique, from the MEP latency recorded during moderate muscular contraction, with an intensity of stimulation of 130% with respect of the rMT. F-waves and peripheral CMAP were evoked with electrical supramaximal stimulations of the right ulnar nerve at wrist. The MEP size was measured as a percentage of supramaximal CMAP size (i.e., the amplitude ratio), which provides a more reliable estimation than the peak-to-peak MEP size [27]. The MEP duration (in ms) was measured from the latency of onset to the return to baseline for both resting and facilitated MEPs [28]. As known, a prolonged MEP duration reflects a temporal dispersion of the cortico-spinal response, thus suggesting a CNS pathology affecting the central motor pathway [27].

The assessment of silent periods (SPs), i.e., contralateral SP (cSP) and ipsilateral SP (iSP), represents the main single-pulse TMS methods for exploring and quantifying the intracortical and intercortical motor inhibitory components, respectively [29]. Moreover, as SPs reflect an index of inhibition of volitional motor activity, rather than a MEP inhibition per se, they are of particular interest for exploring the inhibitory components of the cortico-spinal tract and the interhemispheric correlates of voluntary motor output [27].

When TMS is applied to the M1 contralaterally to the target muscle, the obtained parameter is named cSP [29]. In this case, TMS typically elicits a MEP in the target muscle, followed by a suppression of the voluntary ongoing EMG activity for a period of up to some hundred ms [30]. The cSP is then quantified by its duration, with a longer cSP interpreted as a greater cortical inhibition and shorter duration as a cortical disinhibition [29]. The cSP is generated by both cortical and spinal contribution: the first portion (0–50 ms) is considered to be of spinal origin [30,31], including after-hyperpolarization of motor neurons and recurrent inhibition by the activation of Renshaw cells, or double synaptic inhibition via the Ia inhibitory interneurons [30,31,32,33,34]; the later part (50–200 ms) is attributed to an intracortical inhibition of the cortico-spinal output [30,31,35,36,37]. Since the contribution of cortical mechanisms is considered to be larger (75%) than the spinal ones (25%), the cSP is assumed to reflect the activation of intracortical inhibitory interneurons, mainly by the GABAergic transmission within the M1, particularly by the GABA-B receptors [38,39].

The iSP is evoked by applying TMS to the same hemisphere of a tonically contracting muscle, and, as such, it is viewed as the correlate of transcallosal inhibition [40]. Proposed mechanisms are the following: TMS pulses activate glutamatergic (excitatory) callosal motor fibers synapsing on GABAergic (inhibitory) interneurons in contralateral M1 [41,42]. This would cause a net inhibitory effect and result in a brief depression of the descending cortico-spinal activity that supports the tonic muscle contraction [41,42]. In the contracting muscle, this will appear as a brief suppression or attenuation of the ongoing EMG activity. As for the cSP, the iSP is also quantified by its onset and duration, with greater duration interpreted as a more intense interhemispheric inhibition, and vice versa [29]. Unlike the cSP, the iSP is assumed to be a fully cortical phenomenon: indeed, the iSP does not lower the amplitude of the H-reflex, thus suggesting a lack of any spinal contribution [40].

In the present study, the cSP and iSP were recorded with ~50% of the maximal voluntary tonic contraction of the FDI, evoked by single TMS pulses at 130% of rMT, as recommended [27]. For both recordings, 10 single stimuli were delivered to the contralateral and ipsilateral M1, respectively, and a brief pause (~20 s) was allowed following each stimulus to decrease the possibility to be fatigued. The onset of the cSP and iSP (i.e., their latency) was evaluated for waveform averaged as the temporal interval where the EMG activity dropped ≤75% of the amplitude of the pre-stimulus level. The mean cSP and iSP duration of the rectified trials was considered, with duration measured for all traces as the time from when the EMG-rectified activity dropped ≤75% of the pre-stimulus level to when it returned >75%. This activity level was considered for onset and ending of the cSP and iSP, in order to obtain an objective and reproducible analysis, thus reducing the risk of doubtful interpretation and minimizing bias [29].

A standardized safety checklist was used to screen all individuals [23] and to exclude any neurological disease or medication possibly affecting CNS excitation state. All procedures were performed with participants seated in a dedicated armchair with constant EMG monitoring to guarantee a desirable level of tonic EMG activity during contraction or a total muscular relax. Once collected, data were stored on a dedicated PC by means of ad hoc software that allows one to acquire, process, and analyze data [43]. To reduce the intersubject variability, TMS recordings were executed in the same lab and experimental conditions, at the same time (~11:30 a.m.), and by the same trained operators.

### 2.3. Statistical Analysis

Given the non-normal distribution of most data, non-parametric statistics were adopted. The Mann–Whitney test for independent datasets was used for between group comparisons, followed by the Bonferroni correction for multiple comparisons. In order to avoid missing significant differences due to the relatively low number of individuals recruited, we also calculated the effect size of all differences between patients and controls with the rank-biserial correlation by Wendt (*r* = 1−(2U)/(n1 n2)) [44]. With this approach, an *r* of 0.1 is considered as “small”, 0.3 “medium”, and 0.5 “large”. The Spearman’s rank correlation coefficient was used to evaluate the correlations.

## 3. Results

Table 1 summarizes clinical-demographic and serological features, as well as data from the main diagnostic exams. The right-handedness of all participants was confirmed by the EHI. The general examination was unremarkable in all participants, with the exception of two overweight patients and one underweight patient. Apart from a patient with diffuse and symmetric brisk tendon reflex at upper limbs (without any pathological reflex, including the Hoffmann sign), neurological exams were all normal. Three subjects of the CD group had co-morbidities: autoimmune thyroiditis (one), Raynaud phenomenon (one), and fibromyalgia and psoriasis (one). All subjects were drug-free, except for a patient taking l-thyroxine, with normal levels of thyroid hormones. The two groups were comparable for age, gender, anthropometric features (height, weight, and body mass index), and educational level. Scores at MoCA and HDRS were significantly worse in the CD group than in controls, with a large effect size (Table 2), although the difference of MoCA only remained significant after the Bonferroni correction. Brain CT ruled out intracranial calcifications and clear neuroradiological abnormalities.

The cSP and iSP durations were significantly shorter in CD subjects compared to controls, with a large effect size and also after the Bonferroni correction, whereas no significant difference was found for their latency. Finally, a smaller MEP amplitude was observed in patients than controls, with a large effect size, but not after correction, and with a comparable amplitude ratio between the two groups (Table 2).

**Figure 1:**
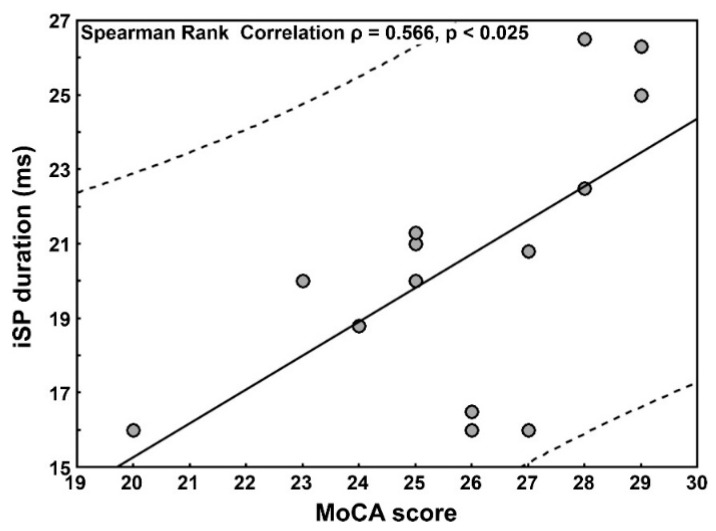
Correlation between MoCA score and iSP duration in patients with celiac disease. Legend: MoCA = Montreal Cognitive Assessment; iSP = ipsilateral silent period; continuous line: linear regression lines; dashed lines: limits within which 95% of observations are expected.

Lastly, correlations between the TMS measures and clinical data that were found to be significantly different (cSP duration, iSP duration, and MoCA) disclosed a significant positive correlation between the MoCA score and iSP duration in patients (Figure 1).

## 4. Discussion

### 4.1. Main Findings

In this cross-sectional study, we have first explored non-invasively and in vivo the interhemispheric functioning to TMS in newly diagnosed patients with CD compared to healthy subjects. First, we have confirmed a pattern of intracortical disinhibition in CD, in terms of a shorter cSP duration, thus further supporting the impairment of GABA-mediated intracortical circuits in non-gluten restricted patients [19]. More importantly, we have found an intercortical motor disinhibition, as indexed by a shorter iSP duration, in these patients, suggesting an electrophysiological involvement of the corpus callosum (CC), which positively correlated with worse cognitive performances in asymptomatic patients. Of note, the iSP is thought to be entirely of cortical origin, without a spinal contribution, such as that described for the cSP [30,31,35,40], thus supporting a cortical localization of this disinhibition. Overall, the pathomechanisms underlying these findings seem to be rather complex, also considering the lack of previous investigations and confirmation.

It is worth mentioning that, to date, the integrity of interhemispheric mechanisms of motor cortex excitability and their correlation with cognitive status has been studied in only few neurodegenerative disorders. In mild-to-moderate Alzheimer disease (AD), iSP latency was significantly longer than in controls, whereas iSP duration did not differ between groups. However, no correlation between iSP latency and cognitive function was noted, suggesting that the intercortical motor inhibition might be independent of cognitive impairment in the mild and moderate stages of AD [46]. In another study in AD, the increased iSP latency was not associated with impaired white matter integrity at diffusion tensor imaging, thus hypothesizing that different physiopathological phenomena can account for the reduced transcallosal inhibition observed in these patients [47]. In more severe AD cases, an increased duration of iSP, along with an early onset latency, has been reported. No correlation was found between cognitive performance and the duration of iSP when the authors mixed both hemispheres, whereas there was a significant negative correlation in the right side if the hemispheres were analyzed separately [48].

In autoimmune and degenerative disorders where the CC is commonly affected, such as MS, the duration of iSP had the highest sensitivity and was not in correlation with MRI-based CC abnormalities in a sample of 49 early patients with relapsing–remitting MS [49]. A subsequent study confirmed that the iSP was altered in early MS and yielded complementary information on subclinical changes. Since pathological brain plasticity has been demonstrated in MS, a compensatory role of the ipsilateral motor and premotor areas was hypothesized [50]. Interestingly, in relapsing–remitting MS patients, the iSP was in correlation with executive cognitive domains, processing speed, visual memory, and physical disability, suggesting that lesioned CC can worsen the level of cognitive impairment and independence status, likely through a “disconnection mechanism” [51].

Finally, in Marchiafava–Bignami syndrome, which is characterized by an early and prominent callosal involvement, a longitudinal clinical, MRI, and TMS study was carried out both in acute stages and six months following the symptoms onset. The baseline assessment demonstrated marked MRI changes, affecting the whole CC. After treatment, symptoms rapidly resolved, along with the neuroradiological changes, except for cognitive impairment. Regarding the iSP, it was not recordable at baseline, whereas it re-appeared at follow-up, also showing a slightly prolonged duration [52].

In the present study, we found an interhemispheric disinhibition, along with a positive correlation between the iSP and MoCA in CD (i.e., a shorter iSP duration with worse MoCA scores). Although the patients were neurologically asymptomatic and their mean MoCA score was still within the normal limits, a statistically significant difference (also after the Bonferroni correction) was found with respect to age- and education-matched healthy subjects. Therefore, these results, although preliminary and in need for further confirmation, might be viewed as an early finding of subclinical cognitive impairment in CD. In this scenario, TMS might identify subclinical changes early and monitor them after the adoption of the GFD.

It is well known that adult subjects with CD may complain some cognitive symptoms, usually in terms of “brain fog”, that improve once the GFD is started, although they may re-appear after incidental gluten intake [53,54]. Difficulties in attention and concentration, lapses in episodic memory and word-retrieval, decreased mental acuity, and episodes of disorientation or “confusion” are commonly reported complaints [55]. In some severely affected cases, even an overt dementia can develop [55,56,57,58]. Nevertheless, most of the previous studies usually included heterogeneous cohorts, at different disease phases, or without controls. In a very recent pilot study [59], both newly diagnosed CD patients and established CD patients underperformed relatively to controls in visual and verbal memory, whereas the established CD group only underperformed in visual–constructive abilities. These findings confirm that cognitive dysfunction in CD may be already present at diagnosis [59], as observed in our sample. Furthermore, a population-based study found that CD patients had the relevant impairment of reaction time and significantly more anxiety, depression, thoughts of self-harm, and health-related unhappiness [60]. In the same population, advanced neuroimaging showed a significantly enhanced axial diffusivity in several brain areas, including CC [60]. Therefore, it is possible that subclinical neurophysiological changes in interhemispheric transmission might be already evident at the disease onset, as also proposed by our study.

In line with these findings, a recent review of the electrophysiological studies in CD [61], including those using TMS [62], seem to converge on a global pattern of “hyperexcitable celiac brain”, that in part improves following a long-lasting GFD. Of note, an overt hyperexcitability is constantly observed in vascular or degenerative dementia [63,64,65]. Since the GFD may exert some neuroprotection, the diet needs to be adopted as soon as possible, though its effects on CNS manifestations (and in particular cognitive features) are debated yet [59]. Translationally, the identification of novel and modifiable risk factors is of pivotal relevance for diagnostic, prognostic, and therapeutic purposes.

Psychiatric co-morbidities, and depression in particular, have been frequently associated with CD [66,67]. In our patients, depressive symptoms were significantly higher than in controls, although the mean raw score was suggestive of a mild depression. Furthermore, the difference observed did not resist after the Bonferroni correction. However, depressive disturbances can substantially affect the quality of life and are a reliable marker of poor adherence to the diet [68]. Screening CD subjects for depressive symptoms is therefore crucial, including follow-up visits, to promptly suggest appropriate pharmacotherapy and/or psychological support. Clinically, improvements can be expected only following a long-lasting gluten restriction (>5 years) [69], thus emphasizing the need for sustained and adherent GFD also on neuropsychiatric symptoms of CD.

In this context, TMS has been used to explore inhibitory and excitatory interactions within motor cortical regions in several neuropsychiatric disorders [70]. Specific TMS protocols also provide insights into the regulation of different neurotransmission systems [71]. For instance, rMT and its changes are regarded as an index of membrane excitability of the cortico-spinal neurons and interneurons within M1 [27]. It is increased by drugs blocking voltage-gated sodium channels [72,73], whereas is not affected by drugs acting on GABA [73], glutamate [74,75], or dopamine [76]. TMS also activates inhibitory cortical circuits containing GABAergic interneurons and, among them, the cSP is known to be influenced mainly by GABA [77]. Similarly, transcallosal inhibition represents the spread of an inhibitory signal from a motor cortex to the other [78]. As such, the iSP is a complex phenomenon, being the duration of transcallosal inhibition dependent on a GABA-mediated inhibition. In the present study, the shortening of both the cSP and iSP in patients, along with the correlation between the MoCA and iSP, may provide hints towards the involvement of central GABAergic transmission and a relationship between TMS-measured GABAergic dysfunction and cognitive performance in CD.

From an electrophysiological perspective, both the cSP and iSP durations are also known to be shorter in MDD, a finding in line with earlier studies of abnormal GABA functioning in the frontal lobe of depressed subjects [79]. Although the patients included in our study did not have MDD, the observed changes in mood in the context of an intracortical and intercortical disinhibition might support the involvement of GABA circuitries within the M1 and CC, respectively [80]. However, a correlation between HDRS scores and SPs was not found, and, therefore, additional investigations should be encouraged to also extend the present data in the brain areas more closely associated with the pathophysiology of mood disorders. Recently, rMT was found to be higher in MDD patients compared with healthy controls, while cSP and iSP were significantly shorter in duration [79]. The authors also observed a positive correlation between scores in the Beck Depression Inventory and the rMT, and a negative correlation with cSP duration, suggesting a global hypoexcitability of both pyramidal cortical neurons (increased rMT) and GABAergic control (shortened SPs) within the dominant M1, which is consistent with previous reports of dysfunctional glutamate and GABA in the frontal cortex in MDD [79].

Lastly, the reason why patients exhibited smaller MEP amplitude compared to the controls (although not significant after the Bonferroni correction) remains quite difficult to explain, with a stochastic effect due to the relatively small sample size not excluded. Theoretically, because a peripheral nerve disease can affect patients with CD [81,82], it might be hypothesized that a reduced MEP amplitude could be caused by a peripheral lesion of the motor axons. However, the lack of clinical findings, along with normal motor nerve excitability and conduction, ruled out this possibility. Moreover, the amplitude ratio, as well as rMT, CMCT, MEP latency and duration, were normal, thus confirming the absence of any significant abnormality along the cortico-spinal tract conductivity.

### 4.2. Limitations

The main limitation, as usually occurs in most studies with TMS, is the relatively small sample size, though the patients were carefully screened and selected, they were homogenous for clinical-serological features and histopathological findings, were all de novo and drug-free, and matched for age and sex with healthy subjects.

Another caveat is that, since TMS provides a functional evaluation of the interhemispheric activities but not of structural changes, a detailed morphological assessment of the cerebral cortex and CC were not performed, thus precluding correlations with neuroimaging data. The same holds true for an extensive neuropsychological battery of tests. Although we have excluded clear neuroradiological abnormalities in all patients, brain CT remains a gross radiological exam, able to detect intracranial calcifications (found in some CD patients) better than MRI, but with quite a low sensitivity and specificity for CC lesions. Therefore, further studies correlating clinical, TMS, and MRI data are needed.

Lastly, although the results have showed some differences in the excitability to TMS between patients and controls, these data should be viewed as only a part of the complex pathophysiological state of the CNS in vivo. Specifically, caution is needed when interpreting these findings as somewhat definitely representative of the status which the TMS variables are able to measure. Therefore, it should be acknowledged that there is ultimately uncertainty over what is precisely being reflected by such differences.

## 5. Conclusions

An intracortical and interhemispheric motor disinhibition to TMS was observed in de novo, neurologically asymptomatic, CD patients, suggesting the involvement of the GABA-mediated cerebral cortex and transcallosal circuitries. Future studies in larger samples and follow-up during dietary regimen will further support and expand these results in CD and other gluten-related CNS diseases.

## Figures and Tables

**Table 1:** Clinical, laboratory, and instrumental features of celiac disease patients.

No.	Age (Years)	Sex	Family History	Clinical Symptoms	Co-Morbidities	Antibodies	Endoscopy	Histopathology
1	55	F	+	Tiredness, dyspepsia, weight loss, iron deficiency anemia	-	tTG, EMA	Scalloped duodenal folds	3c
2	18	F	+	Asthenia, iron deficiency anemia	-	tTG, EMA	Scalloped duodenal folds	3c
3	25	F	+	Tiredness, iron deficiency anemia, dermatological manifestations	-	tTG, EMA	Scalloped duodenal folds	3c
4	18	F	-	Headache, tiredness, belly pain, iron deficiency anemia	-	tTG, EMA	Scalloped duodenal folds	3c
5	29	M	+	- (familial screening)	-	tTG, EMA	Scalloped duodenal folds	3c
6	45	M	-	Tiredness, weight loss, headache, iron deficiency anemia, abdominal pain	-	tTG	Scalloped duodenal folds	3c
7	36	F	-	Headache, tiredness, iron deficiency anemia, vitamin D deficiency weight loss	Autoimmune thyroiditis	tTG, EMA	Scalloped duodenal folds	3c
8	27	F	-	Abdominal pain, diarrhea, tiredness, unsteadiness, weight loss, iron deficiency anemia	-	tTG, EMA	Scalloped duodenal folds	3c
9	35	F	-	Abdominal pain, diarrhea, nausea, iron deficiency anemia, tiredness	-	tTG, EMA	Scalloped duodenal folds	3c
10	44	F	+	Iron deficiency anemia, stipsis and diarrhea, headache, tiredness	Fibromyalgia, psoriasis	tTG	Scalloped duodenal folds	3c
11	45	F	-	Diarrhea, abdominal discomfort, tiredness	Raynaud phenomenon	tTG	Moderate atrophic villi	3b
12	41	F	-	Dyspepsia, iron-deficiency anemia, diarrhea, weight loss, tiredness, diffuse pain	-	tTG, EMA	Scalloped duodenal folds	3c
13	49	F	-	Alternate alvus, dyspepsia, asthenia, tiredness	-	tTG	Scalloped duodenal folds	3c
14	24	F	-	Tiredness, dyspepsia, weight loss, iron deficiency anemia	-	tTG, EMA	Scalloped duodenal folds	3c
15	20	F	-	Tiredness, iron deficiency anemia	-	tTG, EMA	Scalloped duodenal folds	3c

Legend: F = female; M = male; tTG = tissue transglutaminase antibodies; EMA = endomysial antibodies. Classification of histopathology according to the Marsh–Oberhuber grading system [45]: 3a = mild villous flattening; 3b = severe villous flattening; 3c = complete villous flattening; + = positive/present; - = negative/absent.

**Table 2:** Comparison of clinical features and TMS data of both patients and controls.

Variable	Celiac Disease (n = 15)	Healthy Controls (n = 15)	Mann–Whitney		Effect Size
	*Mean ± SD*	*Mean ± SD*	*U*	*p*	*r*
Age, years	34.10 ± 12.03	34.90 ± 9.18	102	NS	0.093
Height, m	1.60 ± 0.08	1.70 ± 0.09	70.5	NS	0.373
Weigh, Kg	57.90 ± 17.38	61.10 ± 8.31	73	NS	0.351
BMI, Kg/m^2^	21.80 ± 5.99	21.80 ± 2.10	80	NS	0.289
Education, years	14.60 ± 3.44	16.20 ± 3.97	69.5	NS	0.382
MoCA	25.80 ± 2.40	28.00 ± 1.00	46	0.0062 *	0.591
HDRS	8.30 ± 6.30	2.90 ± 2.19	50.5	0.01	0.551
rMT, %	37.10 ± 5.58	36.90 ± 6.42	109.5	NS	0.027
cSP duration, ms	87.30 ± 26.85	123.10 ± 29.71	37	0.0019 *	0.671
cSP latency, ms	44.70 ± 3.81	44.10 ± 3.10	104.5	NS	0.071
iSP duration, ms	20.50 ± 3.54	25.50 ± 3.32	33.5	0.0011 *	0.702
iSP latency, ms	32.90 ± 5.84	34.50 ± 4.80	82	NS	0.271
MEP latency, ms	20.00 ± 1.24	20.30 ± 1.56	97.5	NS	0.133
MEP duration, ms (at rest)	12.4 ± 1.42	13.4 ± 2.04	79.5	NS	0.293
MEP duration, ms (active)	15.4 ± 2.43	15.7 ± 1.62	98.5	NS	0.124
CMCT, ms	6.20 ± 0.85	6.50 ± 0.91	88.5	NS	0.213
MEP amplitude, mV	4.50 ± 1.22	5.80 ± 1.65	56	0.02	0.502
CMAP amplitude, mV	19.80 ± 4.19	22.30 ± 6.64	91.5	NS	0.187
CMAP latency, ms	3.40 ± 0.37	4.00 ± 0.76	44	NS	0.609
A ratio (MEP/CMAP)	0.24 ± 0.09	0.28 ± 0.11	74	NS	0.342
F-wave latency, ms	27.00 ± 2.07	28.20 ± 2.83	92.5	NS	0.178
F-wave amplitude, mV	0.10 ± 0.04	0.13 ± 0.06	80.5	NS	0.284
CMCT-F, ms	5.20 ± 1.01	4.80 ± 0.90	85.5	NS	0.240

Legend: A ratio = amplitude ratio; BMI = body mass index; CMAP = compound motor action potential; CMCT = central motor conduction time; CMCT-F = central motor conduction time estimated by means of the F-waves; cSP = contralateral silent period; HDRS = 17-item Hamilton Depression Rating Scale; SD = standard deviation; iSP = ipsilateral silent period; MEP = motor evoked potential; MoCA = Montreal Cognitive Assessment; NS = not significant; rMT = resting motor threshold; TMS = transcranial magnetic stimulation; bold numbers = statistically significant *p* values; * Significant after Bonferroni correction.

## Data Availability

All the data related to this study are available within the manuscript.
